# Basolateral Amygdala Mediates Central Mechanosensory Feedback of Musculoskeletal System

**DOI:** 10.3389/fnmol.2022.834980

**Published:** 2022-02-16

**Authors:** Nian Liu, Botai Li, Lu Zhang, Dazhi Yang, Fan Yang

**Affiliations:** ^1^Huazhong University of Science and Technology Union Shenzhen Hospital, Shenzhen, China; ^2^The Brain Cognition and Brain Disease Institute (BCBDI), Shenzhen Institute of Advanced Technology, Chinese Academy of Sciences, Shenzhen-Hong Kong Institute of Brain Science-Shenzhen Fundamental Research Institutions, Shenzhen, China; ^3^University of Chinese Academy of Sciences, Beijing, China

**Keywords:** basolateral amygdala, mechanosensory, muscle atrophy, bone homeostasis, semaphorin 3A

## Abstract

Musculoskeletal diseases, such as osteoporosis and sarcopenia, are tremendous and growing public health concerns. Considering the intimate functional relationship between muscle and bone throughout development, growth, and aging, muscle provides the primary source of skeletal loading through contraction force. However, significant gaps exist in our knowledge regarding the role of muscle in bone homeostasis and little is known regarding the mechanism through which the central nervous system responds and regulates unloading-induced bone loss. Here, we showed that the basolateral amygdala (BLA) and medial part of the central nucleus (CeM) are anatomically connected with the musculoskeletal system. Unloading-induced bone loss is accompanied by a decrease in serum semaphorin 3A (Sema3A) levels as well as sensory denervation. *In vivo* fiber photometry recordings indicated that the mechanical signal is integrated by the BLA and CeM within 24 h and subsequently regulates bone remodeling. Moreover, chemogenetic activation of BLA*^CaMKII^* neurons mitigates severe bone loss caused by mechanical unloading *via* increased serum levels of Sema3A and sensory innervation. These results indicate that the BLA integrates the mechanosensory signals rapidly and mediates the systemic hormonal secretion of Sema3A to maintain bone homeostasis.

## Introduction

The musculoskeletal system plays an important role in maintaining the function of body locomotion and metabolic homeostasis, and is composed of skeletal muscles, bones, connective tissues, nerves, and blood vessels. Considering the increase in human life expectancy, musculoskeletal diseases have become a growing public health concern and economic burden, as well as a major contributor to chronic disability worldwide (Hirschfeld et al., 2017; Nielsen et al., 2018). Thus, a better understanding of osteoporosis and sarcopenia is required to provide early diagnosis and develop more effective therapeutic interventions.

Muscle and bone are anatomically and physiologically closely related, and pathophysiologically linked to the decline in function associated with aging (Yu et al., 2014; Edwards et al., 2015). Muscle atrophy and bone loss are also associated with a variety of mechanical milieu, including advanced aging, sedentary lifestyle, spinal cord injury, and microgravity (Lang et al., 2004; Trappe et al., 2009; Qin et al., 2010; Steffl et al., 2017; Garrett-Bakelman et al., 2019). In these mechanical contexts, muscle atrophy always precedes bone loss, but also recovers faster than bone after restoration of mechanical loading (Lloyd et al., 2014). Given the wide area of muscle attachments, muscle force is a primary source of skeletal loading and is critical for maintaining bone homeostasis. Nevertheless, as a hypersensitive mechanosensory organ, how decreased skeletal loading affects bone cannot be fully explained by traditional mechanistic theories linking muscle function and bone morphology and the systemic regulation by which muscle enables bone homeostasis remains largely elusive.

The central and peripheral nervous systems regulate bone remodeling through the neuroendocrine and sympathetic nervous systems (Takeda, 2011; Hii, 2012; Dimitri and Rosen, 2017). We previously showed that inhibitory GABAergic neural circuits from bed nucleus of the stria terminalis (BNST) neurons expressing somatostatin to the dorsomedial ventromedial hypothalamus (VMH) nucleus can regulate chronic stress-induced bone loss (Yang et al., 2020). Sensory and sympathetic innervation of the skeleton interacts with afferent and efferent signals from the central nervous system (CNS), affecting many skeletal pathologies, like pain, fracture, osteoarthritis, and tumor progression (Mach et al., 2002; Martin et al., 2007; Brazill et al., 2019). Moreover, semaphorin 3A (Sema3A), a secreted chemotropic cue from the semaphorin family, has been implicated in osteoblast differentiation and bone formation through sensory innervation (Behar et al., 1996; Fukuda et al., 2013). Previous studies have suggested a sensing system is required to achieve bone homeostasis (Fukuda et al., 2013) and the sympathetic nervous system is known to inhibit bone mass accrual (Chen et al., 2019). Therefore, an intriguing question is whether the CNS is engaged in the mechanosensation of skeletal loading and regulates metabolic balance between muscle and bone.

In this study, we evaluated the mechanosensory role of CNS in maintaining homeostasis between muscle and bone. We showed that repulsive mechanical signals were rapidly acquired by the basolateral complex of the amygdala (BLA) and medial subdivision of the central nucleus (CeM). BLA neurons were activated within 24 h following botulinum toxin type A (BTxA) treatment, while CeM neurons were inhibited after muscle atrophy. In addition, activation of BLA*^CaMKII^* neurons accelerated bone formation and mitigated severe bone loss induced by muscle atrophy. Moreover, the BLA regulated bone anabolism through increased sensory innervation mediated by Sema3A, which was significantly correlated with aging and bone mass in clinical respects. Our findings strongly suggest a mechanosensory role of brain-to-bone circuits in maintaining bone homeostasis and provide a possible early diagnosis for musculoskeletal diseases.

## Results

### Crucial Role of Muscle-Derived Mechanical Loading in Maintaining Bone Homeostasis

**FIGURE 1:**
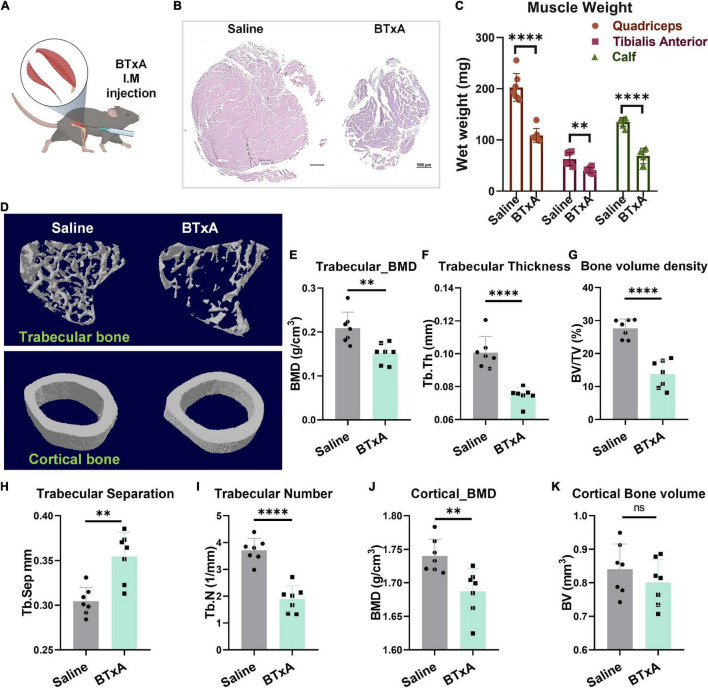
Mechanical role of muscle serves to maintain bone homeostasis. **(A)** BTxA induced muscle-derived force deprivation murine model. **(B)** Left, quadriceps of saline-injected mice; right, quadriceps of BTxA-injected mice. Scale bar, 500 μm. **(C)** Hindlimb muscle mass of quadriceps and calf and tibialis anterior muscles in saline- and BTxA-injected mice. **(D)** Quantitative analysis of representative μCT images of trabecular and cortical bones. **(E–l)** Effect of muscle-derived force on bone parameters; **(E)** trabecular BMD; **(F)** trabecular thickness; **(G)** trabecular volume fraction; **(H)** trabecular separation; **(I)** trabecular number; **(J)** cortical BMD; and **(K)** cortical bone volume. *N* = 7 per group. All data represent as mean ± SD. ^**^P < 0.01, ^****^P < 0.0001, ns means not significant. Student *t-*test for panels **(C–K)**.

To investigate the neural mechanism by which mechanical loading enables bone homeostasis, we recruited an animal model of transient hindlimb muscle paralysis induced by BTxA, which can block muscle-derived force generated by muscle contraction (Figure 1A). Following intramuscular injection of BTxA, our results showed rapid muscle and bone degradation in response to acute muscle paralysis, with significantly decreased muscle wet mass and fiber size (Figures 1B,C) as well as trabecular (−27.3%, *P* < 0.01) and cortical bone mineral density (BMD) (−3.1%, *P* < 0.01) (Figures 1E,J). Trabecular spacing also decreased (−16.4%, *P* < 0.01) in the BTxA-treated hindlimbs compared to the saline-treated mice (Figure 1H). Furthermore, the BTxA-treated mice showed a 25.7% and 48.9% decrease in trabecular thickness and trabecular number, respectively (Figures 1F,I). Trabecular bone showed a significant decrease in bone volume (−47.5%, *P* < 0.0001), but no change was found in cortical bone (Figures 1G,K). Taken together, these data indicate the essential role of muscle function for maintaining bone homeostasis.

### Association of Amygdala With Mechanosensory Feedback From Musculoskeletal System

**FIGURE 2:**
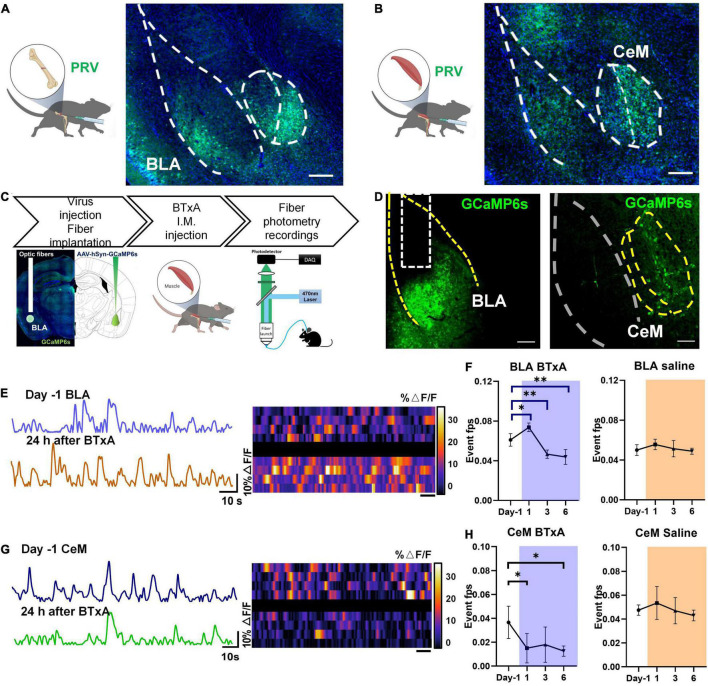
Neural responses to mechanical loading in amygdala. **(A)** Retrograde tracing using pseudorabies virus (PRV) in femur of C57 mice; right, confocal microscopy image of PRV-labeled neurons in basolateral amygdala (BLA) and medial part of central amygdala (CeM). **(B)** Retrograde tracing using pseudorabies virus (PRV) in tibialis anterior of C57 mice; right, confocal microscopy image of PRV-labeled neurons in BLA and central amygdala. **(C)** Experimental procedure of *in vivo* fiber photometry after muscle dysfunction induced by BTxA treatment; **(D)** *in vivo* fluorescence images of AAV9-FLEX-GCaMP6f injected into BLA (left) and CeM (right). Scale bar, 100 μm. **(E)** Examples of fiber photometry in the BLA followed with BTxA i.m. injection within 24 h (left); right, heat map of color-coded changes of fluorescence intensity in the mice as described in panel **(E)**. **(F)** Firing frequency of BLA neurons in response to BTxA i.m. injection or saline within 24 h, 3 days, and 6 days. **(G)** Examples of in fiber photometry in the CeM followed with BTxA i.m. injection within 24 h (left); right, heat map of color-coded changes of fluorescence intensity in the mice as described in panel **(G)**. **(H)** Firing frequency of CeM neurons in response to BTxA i.m. injection or saline within 24 h, 3 days, and 6 days. All data represent as mean ± SD. *N* = 5 (BTxA group), *N* = 4 (saline group); *p < 0.05, ^**^p < 0.01; one-way ANOVA and Tukey comparisons Test were used.

Based on the importance of the CNS and peripheral nervous system in affecting bone remodeling, we next considered how the CNS senses mechanosensory signals from bone after muscle injury or mechanical unloading. We examined the role of the brain-musculoskeletal system axis in the regulation of mechanosensory feedback between bone and muscle. We first mapped synaptic inputs from the musculoskeletal system using engineered rabies viruses (Figures 2A,B). To visualize neurons in the brain with the access to circuits in bone and muscle, we transfected nerve terminals in mice with pseudorabies virus (PRV), which can retrogradely label neural circuit bases of autonomic connections (Smith et al., 2000; Wickersham et al., 2007). The central distribution of PRV-labeled neurons retrogradely traced from bone and muscle was dense in the amygdala. Precisely, the bone nerve terminals were anatomically connected to the BLA, CeM, and piriform cortex (Figure 2A). The muscle nerve terminals were also connected to the posterior part of the BLA, CeM (Figure 2B). These results suggest that the anatomical connectivity between amygdala neurons and musculoskeletal afferent pathways may contribute to the homeostasis of bone and muscle.

To investigate the function of positively labeled brain regions, we assessed the neural activities of amygdala regions in the brain using *in vivo* fiber photometry at different time points, followed by muscle paralysis (Figures 2C,D). In the mouse model of muscle atrophy, the intramuscular injection of BTxA immediately activated BLA neurons within 24 h, as indicated by transduced calcium sensors (Figure 2E). However, the BLA neurons were then inhibited upon BTxA intramuscular injection at day 3, and their activity showed a sustained decline after 6 days of BTxA treatment (Figures 2E,F). Conversely, CeM neurons exhibited sequentially decreased activities within 6 days of muscle contraction inhibition (Figures 2G,H). Blockade of muscle force elicited significant Ca^2+^ responses in BLA neurons on days 1 (20.7%, *P* < 0.05), 3 (−23.6%, *P* < 0.01), and 5 (−28.2%, *P* < 0.01) (Figure 2F), indicating that muscle-derived force could be sensed and transduced by BLA neurons in response to mechanical signals. These results indicate that the neurons in BLA and CeM respond to changes in mechanical signals between muscle and bone.

### Basolateral Amygdala Neurons Integrate Mechanosensory Information From Musculoskeletal System and Regulate Bone Metabolism

**FIGURE 3:**
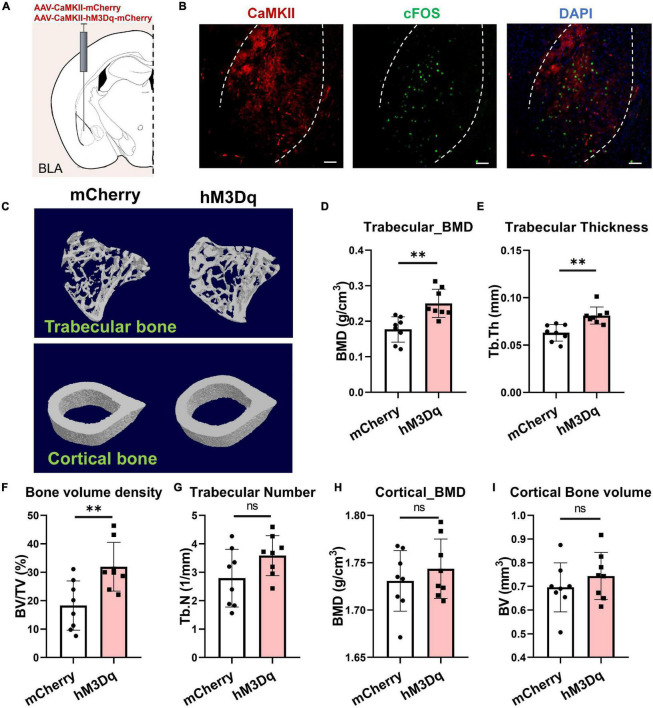
Activation of BLA*^CaMKII^* neurons accelerates bone formation. **(A)** Chemogenetic stimulation of BLA*^CaMKII^* neurons. **(B)** Representative confocal images of cFos+(green) and chemogenetically labeled BLA*^CaMKII^* neurons in BLA CNO i.p. treatment. Scale bar, 50 μm. **(C)** Representative μCT images of trabecular and cortical bones. **(D–I)** Quantitative analysis of μCT images of bone parameters. **(D)** Trabecular BMD, **(E)** trabecular thickness, **(F)** trabecular volume fraction, **(G)** trabecular number, **(H)** cortical BMD, and **(I)** cortical bone volume. *N* = 8 (mCherry group), *N* = 8 (hM3Dq group). ^**^P < 0.01, ns means not significant. All data represent mean ± SD; statistical analysis was assessed using Student *t*-test for panels **(D–I)**.

Previous studies have shown that the BLA contains a large proportion of glutamatergic neurons and a small proportion of inhibitory interneurons (McDonald, 1982; McDonald and Augustine, 1993). Here, we used CaMKII as a marker of excitatory neurons in the BLA, which is restrictedly expressed in glutamatergic neurons (Jones et al., 1994). To investigate the role of BLA*^CaMKII^* neurons in the regulation of bone homeostasis, we bilaterally injected the adeno-associated virus AAV-CaMKII-hM3Dq-mCherry or AAV-CaMKII-mCherry into the BLA. Systemic administration of clozapine *N*-oxide (CNO) was then performed for 4 weeks, and bone parameters were analyzed by X-ray computed microtomography (μCT) (Figure 3A). Results showed that chemogenetic stimulation of BLA neurons promoted bone formation, with a 74.4% increase in trabecular bone volume fraction (BV/TV%) in hM3Dq mice compared to the control mice (Figures 3C,F). Activation of BLA*^CaMKII^* neurons improved trabecular BMD and thickness (41.4% and 28.8%, respectively), but not trabecular number (Figures 3D,E,G). Chemogenetic activation of BLA*^CaMKII^* neurons did not significantly alter cortical BMD or cortical bone volume (Figures 3H,I). Thus, these data highlight the role of BLA*^CaMKII^* neurons in the regulation of bone homeostasis.

### Manipulation of Basolateral Amygdala Neurons Mitigates Muscle Paralysis-Induced Bone Loss

**FIGURE 4:**
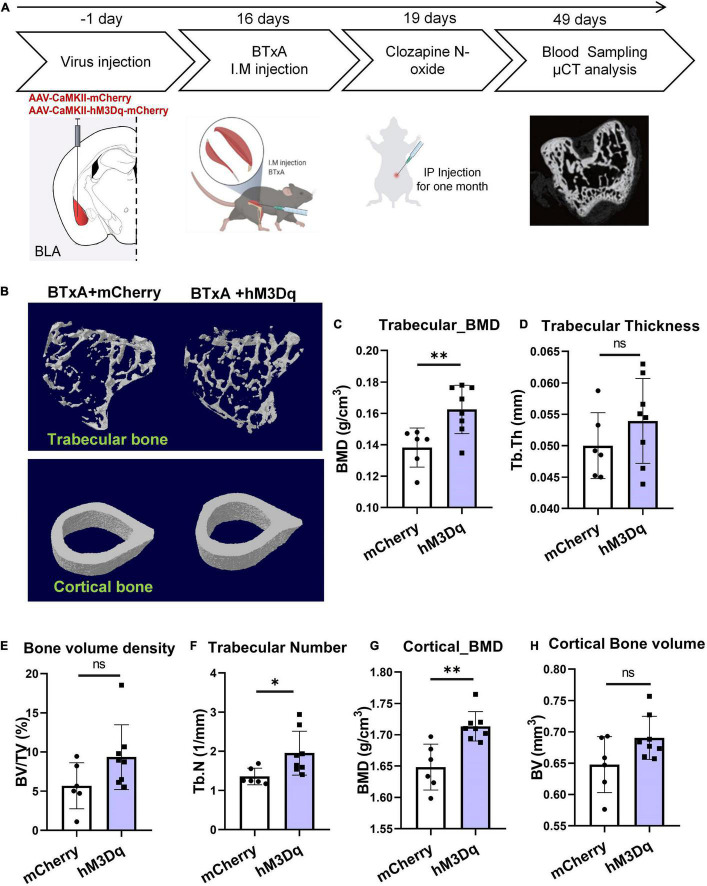
Chemogenetic manipulation of BLA*^CaMKII^* neurons ameliorates severe bone loss induced by muscle dysfunction. **(A)** Experimental procedure of chemogenetic stimulation after muscle dysfunction induced by BTxA treatment. **(B)** Representative μCT images of trabecular and cortical bones. **(C–H)** Quantitative analysis of μCT images of bone parameters. **(C)** Trabecular BMD, **(D)** trabecular thickness, **(E)** trabecular volume fraction, **(F)** trabecular number, **(G)** cortical BMD, and **(H)** cortical bone volume. *N* = 6 (mCherry group), *N* = 8 (hM3Dq group). *P < 0.05, ^**^P < 0.01, ns means not significant. All data represent mean ± SD; statistical analysis was assessed using Student *t*-test for panels **(C–H)**.

We next sought to determine the contribution of BLA neural activity to mechanosensory signals of bone and muscle. We injected AAV-CaMKII-hM3Dq-mCherry or AAV-CaMKII-mCherry bilaterally into BLA regions of C57 mice and then induced muscle atrophy using hindlimb BTxA treatment, followed by intraperitoneal treatment with CNO for 4 weeks (Figure 4A). Results showed that chemogenetic manipulation of BLA*^CaMKII^* neurons ameliorated severe bone loss induced by muscle atrophy (Figure 4B). Based on the μCT results, BTxA treatment significantly diminished trabecular number in the control group (Figure 4F). After 4 weeks of CNO administration, activation of BLA*^CaMKII^* neurons significantly increased trabecular number (43.9%, *P* < 0.05) and BMD (17.6%, *P* < 0.01) in the hM3Dq group compared with the control group (Figures 4C,F). As observed in trabecular bone, cortical bone showed a markedly higher BMD (6.5%, *P* < 0.01) in the BLA-activated mice than in the control group (Figure 4G). Taken together, these results show that BLA*^CaMKII^* neurons can mitigate severe bone loss caused by reduced mechanical unloading after BTxA treatment.

### Basolateral Amygdala Neurons Regulate Bone Remodeling Dependent on Sensory Innervation Mediated by Sema3A

**FIGURE 5:**
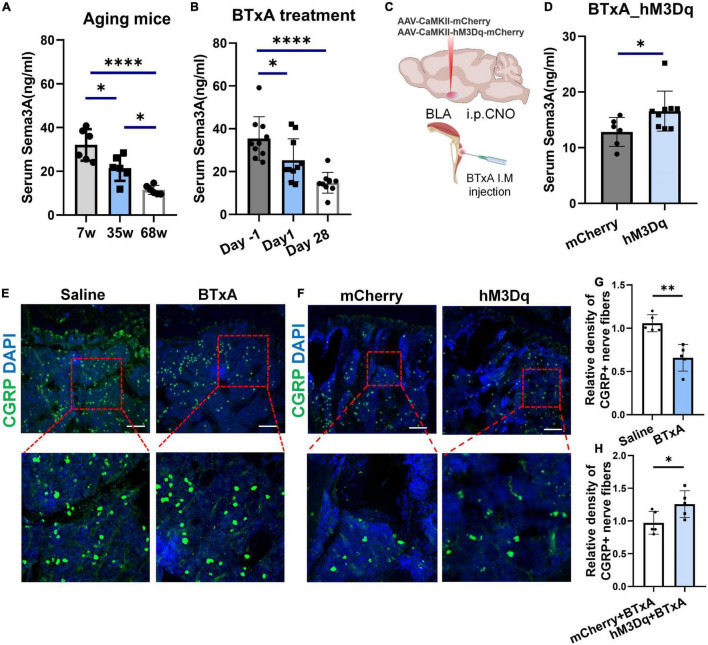
Decreased Sema3A secretion with sensory nerve dysfunction in aging and muscle atrophic mice. **(A)** ELISA of serum Sema3A levels in mice at different ages, i.e., 7, 35, and 68 weeks old. **(B)** ELISA of serum Sema3A levels after BTxA-induced muscle dysfunction. **(C)** Chemogenetic activation of BLA*^CaMKII^* neurons after muscle paralysis. **(D)** ELISA of serum Sema3A levels in BTxA model after chemogenetic activation of BLA*^CaMKII^* neurons. **(E)** Representative immunofluorescent staining images of CGRP (green) in femurs of saline- or BTxA-treated mice. Scale bar, 50 μm. **(F)** Representative immunofluorescent staining images of CGRP (green) in tibia after chemogenetic manipulation of BLA*^CaMKII^* neurons. Scale bar, 50 μm. **(G)** Quantitative analysis of CGRP^+^-nerve fibers in BTxA-injected mouse models. **(H)** Quantitative analysis of CGRP^+^-nerve fibers in hM3Dq vs. mCherry group of BTxA-injected mice. All data represent mean ± SD. Statistical analysis was assessed using student *t*-tests for panel **(D)** and one-way ANOVA followed Tukey comparison test for panels **(A,B)**. *P < 0.05, ^**^P < 0.01, ^****^P < 0.0001.

Sema3A has been implicated to promote bone formation through sensory innervation (Behar et al., 1996; Hayashi et al., 2012; Fukuda et al., 2013) and coordinate with estrogen during bone aging (Hayashi et al., 2019). The loss of bone and muscle occurs more frequently in older people (Hirschfeld et al., 2017). Thus, we wondered whether Sema3A is indispensable for the mechanical coupling of muscle and bone changed with age. To test this, we assessed the serum level of Sema3A in mice at different ages and a significant reduction in Sema3A was observed in aged mice (Figure 5A). Furthermore, we first analyzed the serum level of Sema3A in mice at different time points after muscle paralysis induced by BTxA injection (Figure 5B). Results showed that muscle paralysis led to a significant decrease in serum Sema3A levels, suggesting that Sema3A may play a mechanosensory role between muscle and bone. Notably, a rapid decrease in Sema3A was observed within 24 h of muscle paralysis, suggesting that the association between bone loss and muscle atrophy may be related to Sema3a-mediated sensory innervation (Figure 5B). We also examined the projections of calcitonin gene-related peptide (CGRP) sensory nerve fibers to bone (Figure 5E). Results showed that the density of sensory nerve fibers decreased after BTxA treatment, suggesting that Sema3A-mediated sensory innervation of bone participates in the mechanosensory role of muscle force (Figures 5E,G).

We further assessed changes in serum Sema3A levels after chemogenetic stimulation of BLA neurons through systemic administration of CNO. Results showed a significant increase in serum Sema3A (29.1%, *P* < 0.05) after activation of the BLA neurons in the hM3Dq group compared to the mCherry group (Figures 5C,D). Similarly, immunostaining showed that chemogenetic stimulation of BLA*^CaMKII^* neurons also led to an increase in sensory innervation density in the bone after BTxA treatment (Figures 5F,H). Thus, BLA neurons regulate bone remodeling dependent on sensory innervation mediated by Sema3A. Taken together, these results suggest that muscle strength and loading capacity can interfere with bone homeostasis *via* Sema3A-regulated sensory innervation.

### Clinical Evidence for Role of Sema3A as a Biomarker of Age-Related Muscle and Bone Loss

**FIGURE 6:**
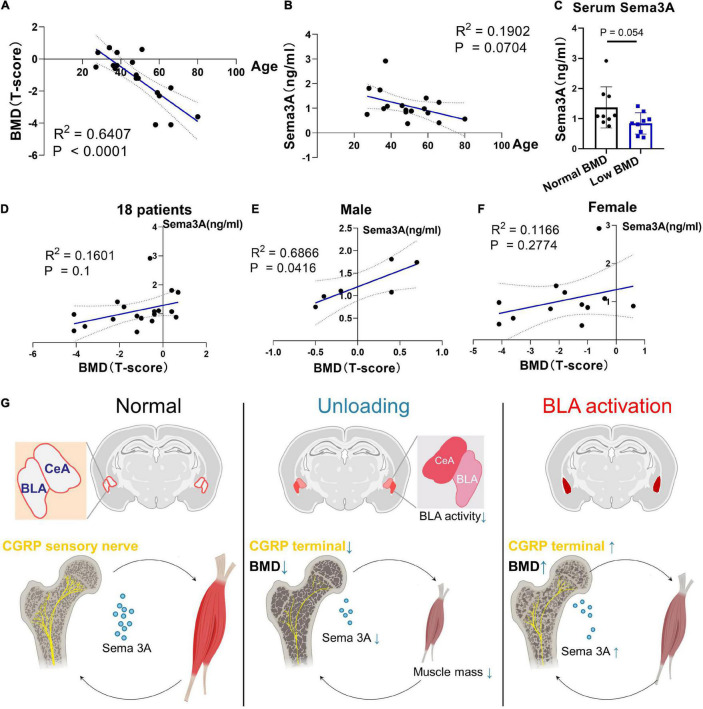
Clinical cases for potential application of Sema3A as a prognostic signal of bone loss. **(A)** Correlation between BMD (T-score) and age (years) in 18 patients. **(B)** Correlation between BMD (T-score) and Sema3A level in 18 patients. **(C)** Serum Sema3A level between normal BMD group (T-score > –1) and low BMD group (T-score ≤ –1). **(D)** Correlation between BMD (T-score) and Sema3A level in 18 patients. **(E)** Correlation between BMD (T-score) and Sema3A level in male group. **(F)** Correlation between BMD (T-score) and Sema3A level in female group. For all clinical data, linear regression models were used to explore the correlation between two variables. *P* < 0.05 was considered significant. **(G)** Basolateral amygdala (BLA) mediates mechanosensory role of bone in response to mechanical unloading.

To address whether the level of Sema3A is associated with prognostic signals of bone loss, we recruited 18 hospitalized patients aged from 27 to 80 years and monitored their BMD using the dual energy X-ray absorption method (DXA), followed by enzyme-linked immunosorbent assay (ELISA) of serum Sema3A levels. Results showed a significant correlation between BMD and age (Figure 6A) as well as decreased serum Sema3A levels with age (Figure 6B). Moreover, patients with osteoporosis (T-score < −2.5) or low bone mass (T-score between −2.5 and −1) had lower serum Sema3A levels compared to the normal BMD group (Figures 6C,D). When sex differences were considered and analyzed separately, a significant correlation between BMD and serum Sema3A was observed in males compared to females (Figures 6E,F). Therefore, we inferred that the secretory pattern of Sema3A, which decreased in individuals with lower BMD, may correlate well with biomarkers of bone remodeling and respond to mechanical unloading. These data suggest that Sema3A may serve as a molecular marker of pre-diagnosed indicators of osteoporosis.

## Discussion

Throughout life, muscle and bone are intimately correlated in structure and function during growth and development. Age-related pathologies of musculoskeletal diseases often occur concurrently, e.g., sarcopenia and osteoporosis, referred to as osteosarcopenia (Reginster et al., 2016; Hirschfeld et al., 2017). Both conditions include a higher risk of falls, fractures, and mortality (Drey et al., 2016; Hirschfeld et al., 2017; Inoue et al., 2021). Muscle is a primary source of mechanical force applied to bone and muscle dysfunction can lead to a reduction in both muscle and bone mass (Reilly and Franklin, 2016). Mechanical unloading can induce muscle atrophy as well as bone loss, such as experienced during spaceflight and prolonged bed rest (Krasnoff and Painter, 1999; Tesch et al., 2005; Lang et al., 2006; Morgan et al., 2012). Currently, however, current studies cannot fully explain how decreased skeletal loading results in a decrease in bone mass. Thus, the question arises as to how the association between low muscle mass and bone density during aging is linked. Here, we showed that age-related bone and muscle loss was accompanied by a reduction in Sema3A levels as well as decreased sensory innervation in the bone. Data from our hindlimb muscle paralysis murine model showed that decreased muscle loading resulted in a severe decline in bone mass, attributed to denervation mediated by Sema3A. Previous studies have shown that bone loss remains severe and bone deterioration is difficult to rejuvenate even after restoration of muscular function and mechanical milieu (Warner et al., 2006; Keyak et al., 2009). This is consistent with our results, which showed that eliminating muscle-derived force led to severe bone loss with unloading (Figure 1). Although the molecular mechanisms that cause both muscle and bone loss remain unclear, clinical data suggest that therapeutics targeting either sarcopenia or osteoporosis alone may not be sufficient to prevent fracture (Hirschfeld et al., 2017). Thus, future studies are necessary to identify and develop new therapeutic approaches that effectively target both bone and muscle. Our data highlight the potential role of sensory nervous system integrity in bone degenerative diseases, such as osteoporosis and sarcopenia.

However, there is also evidence that muscle dysfunction primarily affects bone mass through receptor activator of nuclear factor-κB ligand (RANKL)-mediated osteoclastogenesis in a nuclear factor of activated T cells 1 (NFATc1)-dependent manner, with a significant up-regulation in RANKL at 7 days and elevated osteoclast number at 5 days following muscle dysfunction (Aliprantis et al., 2012). Notably, serial trabecular realignment imaging indicates that initial signaling of bone resorption occurs much earlier than the activation of RANKL-mediated osteoclastogenesis (Aliprantis et al., 2012). Furthermore, significant loss of trabecular bone occurs within the first 24–48 h of muscle dysfunction, before the observed changes in RANKL expression and osteoclast number (Poliachik et al., 2010; Aliprantis et al., 2012). However, we have only just begun to understand how this initial signaling, independent of RANKL-mediated osteoclastogenesis, is sensed, and how the key physiological relationship between bone homeostasis and neuromuscular functions is integrated and transduced. Here, we identified a neuroanatomical loop between the CNS and musculoskeletal system (Figure 6G). Notably, we found that BLA and CeM neurons were anatomically connected to bone and muscle tissues (Figures 2A,B) and muscle paralysis induced by BTxA evoked different responses in the two brain regions: i.e., BLA neurons were activated in response to mechanical unloading, whereas CeM neurons were inhibited 24 h after BTxA injection (Figures 2E–H).

As primary input structures of the amygdala, the BLA and CeA have long been implicated in emotional reactions (fear, anxiety) (LeDoux, 2000; Tye et al., 2011), aversive perceptions (Corder et al., 2019), aggression (Tulogdi et al., 2010; Marquez et al., 2013), escape (Terburg et al., 2018), and defensive behaviors (Tovote et al., 2016; Gu et al., 2020). Previous studies have focused on the broad role of the amygdala in integrating sensory inputs from the thalamus and cortex (Turner and Herkenham, 1991; McDonald, 1998). However, it is not clear whether the BLA and CeA are involved in mechanosensation of the musculoskeletal system. Therefore, it is important to resolve how the BLA processes sensory inputs from coordinated neurons within large neural ensembles during mechanical perception. Here, we showed that the BLA and CeM were anatomically connected with bone and muscle and the mechanical capacity of muscle affected the neural activities of BLA and CeM neurons (Figure 2). Activation of BLA neurons encoded a sensory output and modulated bone homeostasis *via* peripheral sensory innervation in the bone (Figures 5C,D,F). In contrast, the CeA responded negatively to muscle dysfunction, which may provide mechanosensory feedback signals to regulate musculoskeletal system balance. However, considering the robust projections from the BLA to CeA regions, which play multiple roles in anxiety, fear condition, and defensive and appetitive behaviors (Ciocchi et al., 2010; Tye et al., 2011; Kim et al., 2016), we could not rule out the role of BLA-projecting CeA neurons in bone or muscle loss, or the possibility of a hierarchical organization in perception of mechanical forces. In this respect, how intercellular projections transmit mechanosensory signals remains unresolved and requires further study. The CeM also sends projections to the hypothalamus and brainstem, which may serve as an output station to coordinate conditioned autonomic and motor behaviors (LeDoux et al., 1988; Ciocchi et al., 2010; Tovote et al., 2016). As is well-known that defense, escape, and anxiety-related behaviors in animals require the coordination of many bones, joints, and muscles to complete the process of movement. Notably, amygdala networks play a key regulatory role in such defense and locomotion processes (Tulogdi et al., 2010; Tovote et al., 2016; Terburg et al., 2018), suggesting that this brain region favors forming the ability to exercise. As a brain region proposed to be a stress center, BLA has been reported to stimulate the release of bioactive osteocalcin from bone and regulate acute stress response (Berger et al., 2019). Bone-derived osteocalcin is also implicated in the enhancement of muscle function during exercise (Mera et al., 2016). These findings suggest that signaling in the amygdala plays a role in the regulation of musculoskeletal functions. In addition, BLA has been reported to label by viral retrograde tracing from the femur (Denes et al., 2005). Recently, a study from Pahk et al. (2021) has reported that the activities of stress-related amygdala is associated with osteoporosis in postmenopausal women. The metabolic activity of amygdala is much higher in patients with osteoporosis, which is also well correlated with psychological stress level (Pahk et al., 2021). Our data showed that activation of BLA*^CaMKII^* neurons increased trabecular BMD and bone mass under normal physiological conditions (Figure 3). Moreover, BLA*^CaMKII^* neurons also ameliorated the severe bone loss induced by muscle unloading after BTxA treatment (Figure 4). Furthermore, the degradation of both trabecular and cortical bone induced by acute muscle paralysis was mitigated by chemogenetic manipulation of BLA*^CaMKII^* neurons. Taken together, these results consistently suggest that the amygdala acts as both a major sensory interface and a principal output station, capable of sensing or transducing spontaneous mechanical signals in different mechanical milieu, which is necessary for musculoskeletal homeostasis in adult life and aging. Nevertheless, understanding the role of the amygdala in integrating sensory inputs, generating hormonal outputs, and modulating bone metabolism under different mechanical environments can help to clarify the mechanisms leading to the rapid mechanosensation of bone. Our study provides a preliminary understanding of the central neural basis underlying muscle function-mediated bone homeostasis during aging and musculoskeletal diseases.

Many secreted neural factors are pivotal for maintaining muscle and bone homeostasis (Brotto and Bonewald, 2015). Sema3A is implicated in the regulation of bone turnover through sensory innervation (Behar et al., 1996; Fukuda et al., 2013) and coordinates with estrogen during bone aging, with age-dependent reductions in Sema3A contributing to osteocyte survival (Hayashi et al., 2019). Also, the Sema3A/neuropilin axis exhibits an osteoprotective function and mitigates the pathologies of bone fracture healing and osteoporosis (Hayashi et al., 2012; Verlinden et al., 2013; Li et al., 2015). Although peripheral nerves around the bone are well-characterized in the maintenance of bone remodeling (Ducy et al., 2000; Chenu, 2004; Oury et al., 2010), how muscle function affects sensory innervation in the bone and how peripheral nerves sense the bone microenvironment and communicate with specific brain regions remain largely unknown. Based on previous evidence, we further demonstrated that Sema3A was significantly reduced after muscle function impairment, suggesting that it is related to the mechanosensory mechanism of bone (Figure 5B). In addition, our chemogenetic manipulations showed that activation of the BLA regulated the peripheral circulation level of Sema3A, revealing that the BLA plays a critical role in integrating mechanosensory inputs in the CNS (Figures 3, 4). Furthermore, aging is strongly associated with osteoporosis and sarcopenia (Hirschfeld et al., 2017). Interestingly, evidence from human studies shows that the decrease in muscle mass and strength appears to precede bone decline in many cases, e.g., spaceflight or aging (Burr, 1997; Tesch et al., 2005; Morgan et al., 2012; Lloyd et al., 2014). In the current study, serum levels of Sema3A were altered in both muscle paralysis and aging mice (Figures 5A,B), suggesting that muscle dysfunction may be related to the bone protection role of Sema3A. Thus, Sema3A may serve as a sensitive marker of muscle function imposed on bone homeostasis. Notably, evidence from our animal and clinical data strongly suggests the Sema3A may play a role as an early diagnostic indicator of the skeletal system before the occurrence of clinically significant bone loss. In our study, the decreased serum Sema3A level was more often observed in people with osteoporosis or individuals with low bone mass, especially in male patients (Figures 6C,E). Although the investigation of bone and muscle function in female is of great meaning, it requires systematic identification of the impacts of estrogenic secretion on both bone and muscle metabolism. Moreover, we acknowledged the limitations, such as the small number of patients. This pilot study was a necessary reference to evaluate the feasibility of large-scale clinical research in future. Further clinical studies will be needed to investigate fully the therapeutic role of Sema3A on the therapy of musculoskeletal diseases. Our results may help in the development of osteosarcopenia therapies that may anticipate lesions in advance of the severe consequences of falls, fractures, and hospitalizations. Importantly, endocrine factors could serve as sensory markers for the detection and classification of muscle and bone loss.

Collectively, our study suggests that the amygdala is involved in the central mechanosensory feedback of the musculoskeletal system. The initial mechanosensory signals after unloading are immediately integrated by BLA and CeM neurons. Furthermore, activation of BLA neurons regulates bone homeostasis through Sema3A-mediated sensory innervation. Finally, clinical data showed that Sema3A is significantly correlated with age and BMD, and thus could serve as an early signal for musculoskeletal diseases.

## Method Details

### Animals

All experiments steps containing the usage of live animals, or their tissues were conducted in accordance with the national guidelines of China and approved by the IACUC (Institutional Animal Care and Use Committee) of the Shenzhen Institute of Advanced Technology, Chinese Academy of Sciences. C57/6J mice were provided by Beijing Vital River Laboratory Animal Technology Co., China.

Only male mice aged between 8 weeks and 68 weeks (aged mice) were used in this study. All mice were housed in Specific Pathogen Free (SPF) cages in a temperature- and humidity-controlled environment, fulfill the standard of GB14925-2010 Laboratory Animals Requirement of Housing and Facilities, under a repeated 12-h dark–light cycle and had free access to both food and water. Mouse cages were changed twice a week on fixed day, which experiments were not conducted.

### Stereotaxic Surgery

Mice (8–10 weeks old) were anesthetized under 4% isoflurane and 1% isoflurane was used to maintain the anesthesia throughout the whole process. A stereotaxic apparatus (RWD Life Science, Shenzhen, China) was used for stereotactic brain injection. An incision was made to expose the skull and small craniotomies were made with a micro-drill at the regions of interest. Virus (200 nl) was injected to the BLA (ML ± 3.2 mm, AP −1.22 mm, DV −5.05 mm) and CeM (ML ± 2.25 mm, AP −1.20 mm, DV −4.50 mm) at a rate of 50 nl/min using a 10 μl-Hamilton^®^ syringe with ## needle mounted in a nanolitre injector (Nanolitre 2000, World Precision Instruments). The nano-injector was kept in place for 10 min following injections to allow the adequate diffusion of virus solution and to reduce virus backflow during needle withdraw. The incision was closed using tissue adhesive (Vetbond) and mice were provided with antibiotics and analgesics.

### Intramuscular Injection of BTxA

For botulinum toxin (BTXA) injection, a 100 μl-Hamilton^®^ syringe was used to conduct the hindlimb muscle experiment. The procedure was conducted based on the previous study (Warner et al., 2006). Surgical drape and medical tape were applied to secure the mouse body and leg. Three 1 mm^3^ incisions were opened above the quadriceps, tibialis anterior and calf muscles, then BTxA (2.0 U/100 g) (HengLi, Lanzhou) or saline was injected into these three targeted muscle parts slowly and kept in place for 10 min. After the injection, the wound was carefully closed with Vetbond tissue adhesive (3 M, 1469SB). For Animal behavioral tests, motion evaluation after BTX injection was quantified on days 1, 3, and 7 and weekly thereafter using the assessment of gait disability based on the previous study (Warner et al., 2006).

### Retrograde Neural Tracing From Musculoskeletal System

Polysynaptic retrograde tracing was conducted with modulated pseudorabies virus vector (PRV-CAG-EGFP) (1.3 × 10^10^ PFU/ml), which is a recombinant PRV strain with Bartha background. The virus was purchased from the Brain Case (Cat No. BC-PRV-531). The PRV-Bartha strictly spreads only from infected post- to pre-synaptic neurons in injected sites. Briefly, mice were anesthetized under 4% isoflurane and 1% isoflurane was used to maintain the anesthesia throughout the whole process. For retrograde tracing, 2 μl of PRV was injected into two sites of femoral bone marrow with 10 μl Hamilton syringe. 2 μl of PRV was injected into three sites of tibialis anterior. The needle was kept in place for 5 min to avoid the reflux. Then, swabbed each injection site to minimize non-specific viral spread. To examine the expression of PRV transport to the central nervous system following bone and muscle injections, mice were sacrificed from day 5 to day 7 after postinoculation according to the reference (Denes et al., 2005) and brain sections were examined for GFP expression.

### Fiber Photometry Recording

For *in vivo* fiber photometry, rAAV9-syn-GCamP6f (1.15 × 10^13^ copies per ml) was unilaterally injected into the BLA or CeM. After at least 2 weeks recovery, a unilateral fiber cannula (200 μm core diameter, 0.37 NA, Newdoon Inc.) was implanted into BLA/CEA after virus injection. After the surgery, mice were transferred into their home cage. 4 weeks later, mice were handled 3 times before free-moving recording using Thinker technology photometry system (Spectral channel 500–543 nm for GCamP6). Then muscles of right leg were injected with BTxA as described above. Four timepoints (−1 day, 1 day, 3 days, 6 days) of calcium signal were recorded after the BTxA injection. All Data were processed and analyzed by Thinker software based on Matlab 2017a offline and Prism. The plot data of △*F*/*F* was calculated in accordance with equation:


$$\frac{△\,F}{F_{0}}=\frac{V\,s\,i\,g\,n\,a\,l-F_{0}}{F_{0}-V_{o\,f\,f\,s\,e\,t}}$$

$$F_{0}=\bar{V_{b\,a\,s\,a\,l}}$$

$\bar{V_{b\,a\,s\,a\,l}}$: average value of V_*basel*_ in reference time

*Vsignal*: raw signal value

*V*_*offset*_: baseline value of the system.

### Immunohistochemistry and Microscopy

Animals were anesthetized with sodium pentobarbital, and perfused with ice-cold 4% paraformaldehyde (PFA) in PBS. The samples of brains and legs were fixed overnight in 4% PFA. Then, samples were dehydrated in 30% sucrose for 2 days. The femurs and tibias were decalcified in 10% EDTA solution for at least 14 days. Brains were coronally sectioned at 40 mm thickness. Bone samples were sectioned at 200 mm thickness. The sections were washed with PBS and blocked in 10% normal goat serum (NGS) in PBS with 0.1% Triton X-100 (PBST) for 1 h. Primary antibody was incubated in 0.1% PBST with 1% NGS overnight at 4°C. Primary antibodies used in this study were: rabbit anti-cFos (Cell signaling, #2250, 1:200), mouse anti-CGRP (Abcam, 81887, 1:500). The following day, the sections were washed three times for 10 min in 0.1% PBST and then incubated in secondary antibody. Sections were then washed and mounted with DAPI Fluoromount-G (SouthernBiotech, 0100-20). Secondary antibodies used in this study were: Goat anti rabbit Alexa Fluor 488/594 (Jackson laboratory, 111-547-003/111-587-003, 1:200), Goat anti mouse Alexa Fluor 488/594 (Jackson laboratory, 115-547-003/115-587-003, 1:200). For higher resolution imaging, Zeiss LSM 800 confocal microscope was used.

### Chemogenetic Manipulation

Virus vectors were injected by stereotaxic apparatus as described above. rAAV was produced and purified in house with following titers: AAV9-CaMKII-mCherry (1.2 × 10^13^ vg/mL) and AAV9-CaMKII-hM3Dq-mCherry (5.6 × 10^12^ vg/mL). For the chemogenetic activation of in BLA*^CaMKII^* neurons, AAV- CaMKII-hM3Dq-mCherry (200 nl) was bilaterally injected into the BLA. Control mice also received viral injections expressing mCherry (AAV9-CaMKII-mCherry). The hM3Dq is modified from human M3 muscarinic receptor, which is also called as Designer Receptor Exclusively Activated by Designer Drugs (DREADD), and is specifically activated by clozapine *N*-oxide (CNO). To activate DREADD-expressing neurons in the BLA, 1 mg/kg CNO (Sigma-Aldrich) was intraperitoneally (i.p.) administered to C57 mice at 48-h intervals for 4 weeks. Mice in all groups were injected with CNO to control for the potential effects of CNO. For c-Fos evaluation, an additional CNO injection was administered 90 min before euthanasia.

### Hematoxylin and Eosin Staining (H&E Stain)

Tissues were made into 5-μm paraffin sections for H&E staining. Paraffin sections were dewaxed in xylenes and rehydrated in gradient ethanol (100%, 95%, 75%) each for 2 min. The sections were rinsed in tap water and stained with Hematoxylin (Sigma Aldrich) solution for 1 min, and then blued by running tap water followed by differentiation with 0.3% acid alcohol. Next, the sections were stained with eosin Y solution (Sigma Aldrich) for 30 s followed by dehydration with graded alcohol and clearing in xylene. The slides were then mounted by Eukitt^®^Quick-hardening mounting medium (Sigma Aldrich) for further examination.

### ELISA Analysis

The serum samples were collected using a serum separator tube and centrifuged for 20 min at approximately 1,000 × *g*. The supernatant is collected for assaying. Mouse serum Sema3A was measured with Sema3A ELISA (enzyme-linked immunosorbent assays) Kit (CUSABIO, CSB-EL020980MO), according to manufacturer’s instructions. Human Sema3A s was measured with human Sema3A ELISA Kit (CUSABIO, CSB-E15913h), according to manufacturer’s instructions. The results were analyzed by a Nano Quant plate reader (Tecan, Infinite 200Pro).

### Micro CT Analyses

Mice femora and tibiae were harvested followed by 4% PFA fixation overnight before micro-CT scanning (SkyScan, Model 1065, Bruker). Scanning was performed using the following settings: isotropic voxel 11.53 μm, voltage 48 kV, current 179 μA, and exposure time of 1,800 ms. Three-dimensional (3D) reconstruction was conducted using SkyScan NRecon software (version 1.6.8.0, SkyScan) with a voxel size of 8.66 μm. Datasets were reoriented using DataViewer (version 1.4.4.0, SkyScan), while the calculation of morphological parameters was carried out with the CTAn software (version 1.13.2.1, SkyScan). The 3D reconstructed models were displayed by CTVol software (version 2.2.3.0, SkyScan).

The trabecular bone was analyzed from 0.1 mm of the distal tibial growth plate to 0.9 mm downward. The region of interest was defined according to coronal section image. The trabecular bone volume fraction (BV/TV, %), trabecular thickness (Tb. Th, 1/mm), trabecular number (Tb. N, 1/mm), trabecular separation (Tb. Sp, mm) and BMD (g/cm3) were analyzed from the three-dimensional analysis. The cortical bone was analyzed from proximal growth plate 6.5–7.1 mm. The cortical bone volume fraction (BV, m^3^) and BMD, g/cm3 were analyzed from the three-dimensional analysis.

### Clinical Investigations

In this study, a total of 18 hospitalized patients from 27 to 80 years of age was recruited. All these patients underwent health examination. Patients with the cancer, hyperlipemia, hypertension, hyperuricemia, secondary osteoporosis or who had irregular available to oral anti-osteoporosis drugs (e.g., FOSAMAX) within 3 months before hospitalization were excluded. The bone mineral density of patients was monitored using Dual-emission X-ray Absorptiometry (DXA) (ASY-0049, Hologic, United States). The bone mass level of patients was defined as the T-score in the results of DXA scans analysis. The criteria were classified as normal (T-score > −1), osteopenia (–1 ≥ T-score > –2.5), or osteoporosis (T-score ≤ –2.5). The blood serum was extracted and centrifuged from fresh blood and then stored at −80°C. The serum Sema3A level was detected using Sema3A ELISA kit (CUSABIO, CSB-E15913h) which have been described previously in this study. All procedures involving human participants were approved by the Ethics Committee of the Huazhong University of Science and Technology Union Shenzhen Hospital. Informed consent was obtained from all participants.

### Statistics

Statistical analyses and linear regressions were performed using either GraphPad Prism software (GraphPad Software Inc.) or SPSS 26 (IBM). Data are presented as means ± standard deviation (SD). Normality was determined using the D’Agostino–Pearson normality test. Comparisons between two groups were analyzed using unpaired *t*-test. One-way ANOVA was used for the comparisons among multiple groups. For clinical data analysis, linear regression models were fit to explore the correlation of Sema3A level to BMD (T-score). *P* < 0.05 was considered significant. Statistical significance was set at *P < 0.05, ^**^P < 0.01, ^***^P < 0.001, ^****^P < 0.0001.

## Data Availability Statement

The original contributions presented in the study are included in the article/supplementary material, further inquiries can be directed to the corresponding author/s.

## Ethics Statement

The studies involving human participants were reviewed and approved by Ethics Committee of the Huazhong University of Science and Technology Union Shenzhen Hospital. The patients/participants provided their written informed consent to participate in this study. The animal study was reviewed and approved by Institutional Animal Care and Use Committee of the Shenzhen Institute of Advanced Technology, Chinese Academy of Sciences (CAS).

## Author Contributions

FY and DY supervised the study. NL designed the experiments. NL and BL conducted chemogenetic experiments, animal behavior studies, serum biochemical analysis, *in vivo* fiber photometry, and data analysis. LZ performed PRV retrograde tracing experiments. All authors contributed to the article and approved the submitted version.

## Conflict of Interest

The authors declare that the research was conducted in the absence of any commercial or financial relationships that could be construed as a potential conflict of interest.

## Publisher’s Note

All claims expressed in this article are solely those of the authors and do not necessarily represent those of their affiliated organizations, or those of the publisher, the editors and the reviewers. Any product that may be evaluated in this article, or claim that may be made by its manufacturer, is not guaranteed or endorsed by the publisher.
